# Depletion of Macrophages Improves Therapeutic Response to Gemcitabine in Murine Pancreas Cancer

**DOI:** 10.3390/cancers12071978

**Published:** 2020-07-20

**Authors:** Soeren M. Buchholz, Robert G. Goetze, Shiv K. Singh, Christoph Ammer-Herrmenau, Frances M. Richards, Duncan I. Jodrell, Malte Buchholz, Patrick Michl, Volker Ellenrieder, Elisabeth Hessmann, Albrecht Neesse

**Affiliations:** 1 Department of Gastroenterology, Gastrointestinal Oncology and Endocrinology, University Medical Center Göttingen, 37075 Göttingen, Germany; soeren.buchholz@stud.uni-goettingen.de (S.M.B.); robert.goetze@med.uni-goettingen.de (R.G.G.); shiv.singh@med.uni-goettingen.de (S.K.S.); christoph.herrmenau@med.uni-goettingen.de (C.A.-H.); volker.ellenrieder@med.uni-goettingen.de (V.E.); elisabeth.hessmann@med.uni-goettingen.de (E.H.); 2 Cancer Research UK Cambridge Institute, The University of Cambridge, Li Ka Shing Centre, Cambridge CB2 1TN, UK; Fran.Richards@cruk.cam.ac.uk (F.M.R.); Duncan.Jodrell@cruk.cam.ac.uk (D.I.J.); 3 Department of Medicine, Division of Gastroenterology, Endocrinology and Metabolism, Philipps University Marburg, 35037 Marburg, Germany; malte.buchholz@staff.uni-marburg.de; 4 Department of Internal Medicine I, Martin-Luther-University of Halle-Wittenberg, 06120 Halle, Germany; patrick.michl@uk-halle.de

**Keywords:** pancreatic cancer, drug delivery, macrophages, chemoresistance

## Abstract

Background: The tumor microenvironment (TME) is composed of fibro-inflammatory cells and extracellular matrix (ECM) components. However, the exact contribution of the various TME compartments towards therapeutic response is unknown. Here, we aim to dissect the specific contribution of tumor-associated macrophages (TAMs) towards drug delivery and response in pancreatic ductal adenocarcinoma (PDAC). Methods: The effect of gemcitabine was assessed in human and murine macrophages, human pancreatic stellate cells (hPSCs), and tumor cells (L3.6pl, BxPC3 and KPC) in vitro. The drug metabolism of gemcitabine was analyzed by liquid chromatography–tandem mass spectrometry (LC–MS/MS). Preclinical studies were conducted using Kras^G12D^;p48-Cre and Kras^G12D^;p53^172H^;Pdx-Cre mice to investigate gemcitabine delivery at different stages of tumor progression and upon pharmacological TAM depletion. Results: Gemcitabine accumulation was significantly increased in murine PDAC tissue compared to pancreatic intraepithelial neoplasia (PanIN) lesions and healthy control pancreas tissue. In vitro, macrophages accumulated and rapidly metabolized gemcitabine resulting in a significant drug scavenging effect for gemcitabine. Finally, pharmacological TAM depletion enhanced therapeutic response to gemcitabine in tumor-bearing KPC mice. Conclusion: Macrophages rapidly metabolize gemcitabine in vitro, and pharmacological depletion improves the therapeutic response to gemcitabine in vivo. Our study supports the notion that TAMs might be a promising therapeutic target in PDAC.

## 1. Introduction

Pancreatic ductal adenocarcinoma (PDAC) exhibits an extensive desmoplastic reaction that is characterized by abundant extracellular matrix components, numerous inflammatory cells and cancer-associated fibroblasts (CAFs) [1]. It is undisputed that the tumor microenvironment (TME) closely interacts with surrounding tumor cells via direct physical interaction and multiple signaling cues, promoting tumor progression and therapeutic resistance in PDAC [2]. However, recent evidence points towards a more complex role of distinct components of the TME, with both tumor-restraining as well as tumor-promoting properties [3,4,5,6]. Indeed, several large clinical trials using anti-stromal agents (e.g., hyaluronidase PEGPH20, sonic hedgehog inhibitors, matrix metalloproteinase (MMP) inhibitors) have recently failed to achieve meaningful clinical response rates in PDA patients, and so far, no anti-stromal therapies have been approved [7].

Therefore, in light of the urgent need for novel therapeutic targets in PDAC, it is important to investigate different cell types within the TME regarding its role in mediating therapeutic resistance [8,9].

We have recently shown that CAFs accumulate gemcitabine metabolites intracellularly, a mechanism that may contribute to the failure of this drug by scavenging active gemcitabine metabolites that are not available for tumor cells anymore [10]. Recent single-cell analysis in human and mouse PDAC not only revealed several subsets of CAFs such as inflammatory CAFs (iCAFs) and myofibroblastic CAFs (myCAFs), but also provided compelling evidence that CAFs only make up a small proportion of all cells within the TME [11]. In contrast, immune cells are much more abundant in PDAC and might also contribute to drug scavenging upon gemcitabine treatment. Tumor-associated macrophages (TAMs) make up a large subset of immune cells in PDAC, and pharmacological depletion was shown to reduce metastasis formation in genetically engineered mice [12]. Few studies have reported synergism of TAM depletion and immunotherapies in PDAC [13,14]. In addition, systemic TAM depletion can impede pancreatic tumorigenesis and regress established tumors [15,16]. Although there are some examples of TAMs inhibiting sensitivity to chemotherapy [17,18,19], many mechanisms remain largely unknown.

To assess the contribution of TAMs to gemcitabine resistance in PDAC, we have employed genetic and pharmacological approaches in vitro and in vivo using a liquid chromatography–mass spectrometry/mass spectrometry (LC–MS/MS) assay [20,21,22], the most sensitive method to quantify gemcitabine metabolites in cells and tissue biopsies.

## 2. Results

### 2.1. Gemcitabine Concentration is Increased in Murine Pancreatic Tumors Compared to Pancreatic Intraepithelial Neoplasia and Normal Pancreas Tissue

**Figure 1:**
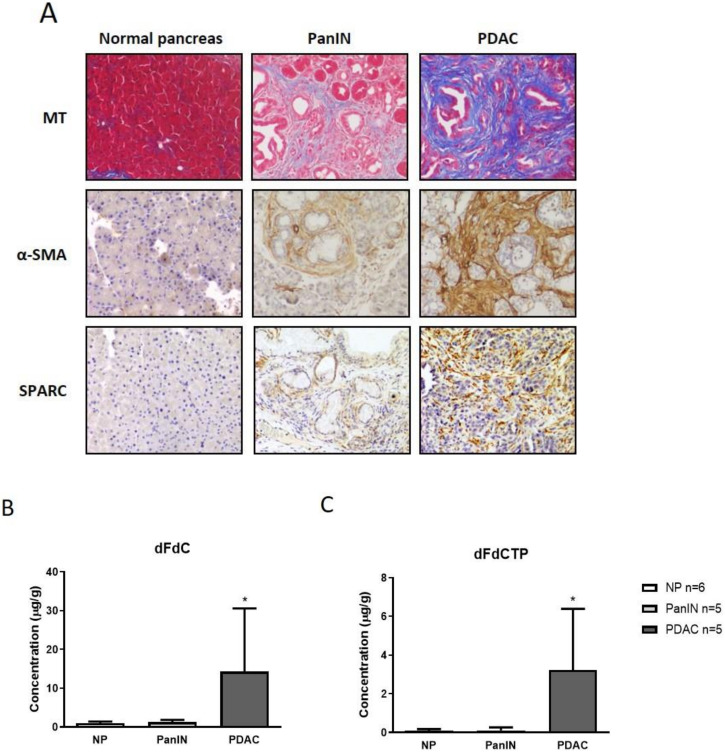
(**A**) Representative Masson trichrome (MT) and immunohistochemical stainings for alpha-smooth muscle actin (α-SMA) and secreted protein acidic and rich in cysteine (SPARC) in normal murine pancreas, murine pancreatic intraepithelial neoplasia (PanIN) and pancreatic ductal adenocarcinoma (PDAC) tissues from *LSL-Kras^G12D/+^*; p48*-Cre* (KC) mice showing progressive desmoplastic features. (**B**,**C**) KC mice and control B6 mice were treated with one dose of gemcitabine at 100 mg/kg intraperitoneally. Tumor tissue (*n* = 5 mice), PanINs (*n* = 5 mice) and normal pancreas (NP; *n* = 6 mice) were assessed for gemcitabine metabolites 2 h later by liquid chromatography–mass spectrometry/mass spectrometry (LC–MS/MS). Native gemcitabine (dFdC) and the active form of gemcitabine 2’,2′-difluorodeoxyuridine-5′-triphosphate (dFdCTP) are significantly increased in tumor biopsies compared to PanINs and normal pancreas tissue (*p* < 0.05, Mann–Whitney U test).considerably older than PanIN-bearing KC mice, age related effects might bias the pharmacokinetic results obtained by LC–MS/MS. However, analysis in tumor-bearing KPC mice of different age showed no correlation of age and intratumoral gemcitabine accumulation (data not shown).

Considering our previous observation that CAFs are capable of metabolizing gemcitabine metabolites [10], and the hypothesis that other stromal cells such as immune cells may also scavenge gemcitabine, we reasoned that the total amount of gemcitabine metabolites should be higher in pancreatic tumor tissue compared to normal pancreas tissue. To test this hypothesis, we investigated gemcitabine delivery in normal pancreas tissue, pancreatic intraepithelial neoplasia (PanIN) lesions and desmoplastic tumors of genetically engineered mice (GEMMs) to compare drug accumulation during tumor evolution. The *LSL-Kras^G12D/+^*; p48*-Cre* (KC) mouse model develops PanIN lesions at 3–4 months of age that progress to invasive PDAC after a latency of 12–15 months [23]. Progression to PDAC is accompanied by the development of a pronounced TME in which acellular (e.g., collagen, hyaluronic acid) and cellular components such as macrophages and CAFs increasingly accumulate. Therefore, we employed the KC mouse model at different stages of pancreatic carcinogenesis (Figure 1A) and administered a single dose of 100 mg/kg gemcitabine.

Bulk tissue from normal pancreata (*n* = 6 mice), PanINs (*n* = 5 mice) and murine PDAC (*n* = 5 mice) were collected 2 h after gemcitabine administration according to previously established and validated protocols and subjected to LC–MS/MS analysis [21,24]. Strikingly, the concentration of native gemcitabine 2′,2′-difluorodeoxycytidine (dFdC) as well as the activated form of gemcitabine 2′,2′-difluorodeoxycytidine-5′-triphosphate (dFdCTP) was significantly elevated in bulk tumor biopsies compared to PanIN tissue and normal pancreas biopsies (Figure 1B,C; *p* < 0.05), suggesting an overall increased uptake of gemcitabine in tumor tissue versus normal tissue.

### 2.2. Murine and Human Macrophages Reduce Cytotoxicity of Gemcitabine In Vitro

**Figure 2:**
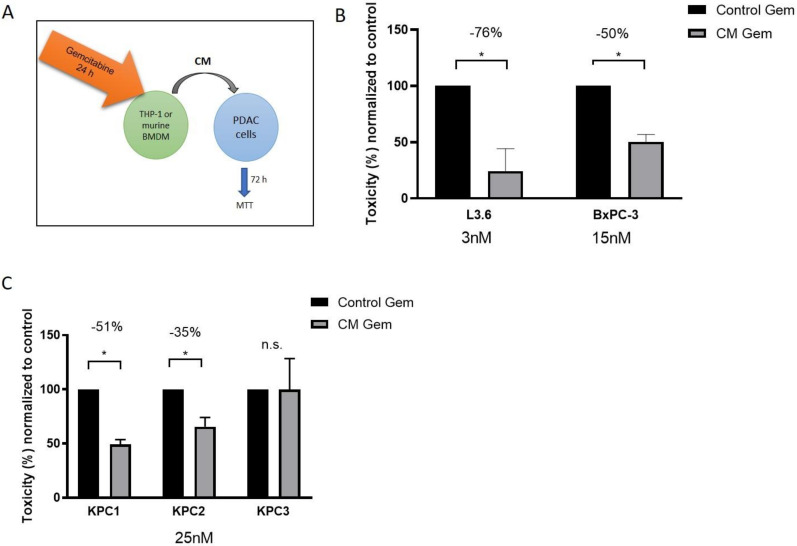
(**A**) Schematic of the conditioned media experiments showing that gemcitabine is first incubated with either THP-1 cells or murine bone marrow-derived macrophages (BMDM) for 24 h before conditioned media (CM) is used for subsequent viability assays on human and murine tumor cells. (**B**) 72 h MTT assay with CM of unpolarized THP-1 cells pre-incubated for 24 h with gemcitabine in L3.6pl (3 nM) and BxPC3 (15 nM) shows a robust decrease in toxicity compared to THP-1 control media with fresh gemcitabine prior to 72 h treatment (*p* < 0.01). Gemcitabine concentrations are adapted because of specific GI_50_. (**C**) An equivalent assay with murine bone marrow-derived macrophages (BMDM) and three KPC cell lines shows a significant decrease in toxicity in two out of three cell lines (KPC-1+2: *p* < 0.01, KPC-3: *p* > 0.05).

Macrophages are abundantly present in the fibro-inflammatory TME of KC and KPC mice, and were shown to correlate with worse prognosis in PDAC [25,26]. To investigate gemcitabine metabolism in macrophages, we used the human monocyte cell-line THP-1. To test whether THP-1 cells mediate chemoresistance towards pancreatic cancer cells in vitro, two human pancreatic cancer cell lines L3.6pl and BxPC3 were treated with supernatant from THP-1 cells that were pre-incubated with gemcitabine for 24 h. As control THP-1 supernatant with freshly supplemented gemcitabine was used (Figure 2A). Supernatant from pre-incubated THP-1 cells led to a significant decrease in cell toxicity in both tumor cell lines (76% decrease for L3.6pl and 50% decrease for BxPC3) compared to controls (*p* < 0.05; Figure 2B). We next sought to determine if murine macrophages derived from bone marrow (BMDMs) of B6 mice are also able to impede therapeutic response in KPC tumor cell lines. Indeed, two out of three KPC tumor cell-lines showed decreased toxicity (51% for KPC-1 and 35% for KPC-2) following 24 h pre-incubation with gemcitabine containing macrophage media compared to THP-1 supernatant with fresh gemcitabine (KPC-1+2: *p* < 0.05; KPC-3: *p* > 0.05; Figure 2C). Notably, THP-1 conditioned media or murine BMDM conditioned media alone did not affect cell viability in human and murine PDAC cells (Supplementary Figure S1). Furthermore, incubation of gemcitabine in cell culture media without THP-1 cells for up to 24 h did not affect the toxicity of gemcitabine compared to freshly administered gemcitabine, indicating that the gemcitabine metabolism of THP-1 cells might be causative for the observed reduction in gemcitabine cytotoxicity in murine and human tumor cells (Supplementary Figure S2).

### 2.3. Macrophages Rapidly Metabolize and Inactivate Gemcitabine

**Figure 3:**
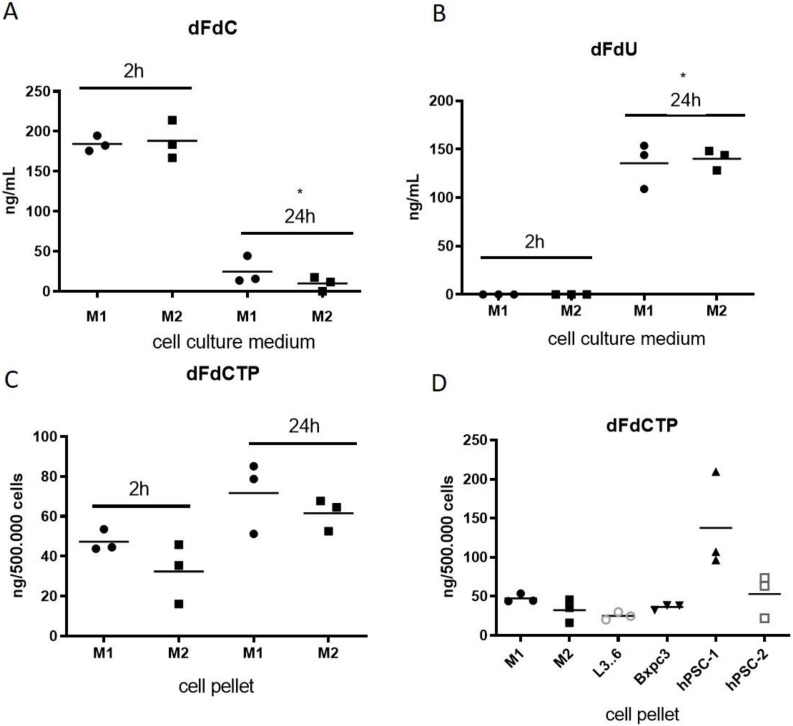
(**A**,**B**) Pharmacokinetic profile of cell culture supernatant from M1 and M2 THP-1 human macrophages following incubation with 1µM gemcitabine for 2 h and 24 h. LC–MS/MS analysis in cell culture supernatant for native gemcitabine (dFdC) (M1: *p* < 0.001 M2: *p* < 0.001) and the deaminated form dFdU shows rapid metabolization of dFdC to dFdU within 24 h. (M1: *p* < 0.001, M2: *p* < 0.0001) (**C**) The active form of gemcitabine (dFdCTP) was determined by LC–MS/MS in cell pellets from THP-1 cells. (M1: *p* > 0.05 M2: *p* < 0.05). (**D**) LC–MS/MS analysis (2 h) for the active gemcitabine metabolite dFdCTP in THP-1 macrophages (M1, M2), human pancreatic cancer cell lines (L3.6pl; BxPC3) and two human PSCs (hPSCs).

To mechanistically investigate the observed effect, we aimed to assess gemcitabine drug uptake in THP-1 cells including the M1- and M2 subtypes. To this end, we polarized cells to generate either M1 or M2 THP-1 cells. Polarization was successfully verified by markers such as CXCL10 (M1-specific), CCL22 (M2-specific) and MRC1 (M2-specific) (Supplementary Figure S3). We treated cultured M1 and M2 THP-1 cells with 1 µM gemcitabine. Cell culture supernatants and cell pellets were obtained after 2 h and 24 h for analysis by LC–MS/MS. Strikingly, the amount of native gemcitabine dFdC was significantly reduced in the media after 24 h of incubation (M1: *p* < 0.001, M2: *p* < 0.001) whereas the deaminated metabolite 2′,2′-difluorodeoxyuridine (dFdU) significantly increased within 24 h (M1: *p* < 0.001, M2: *p* < 0.0001; Figure 3A,B). Conversely, the active, cytotoxic form of gemcitabine, dFdCTP accumulated intracellularly after 24 h of incubation with gemcitabine (M1: *p* > 0.05, M2 *p* < 0.05) (Figure 3C). These experiments provide evidence that macrophages rapidly metabolize gemcitabine thus potentially acting as drug scavengers in the TME of PDAC. Next, we aimed to compare the gemcitabine uptake in cultured M1 and M2 THP-1 cells, BxPC3 and L3.6pl pancreatic cancer cells, and two human pancreatic stellate cell lines (hPSC-1, hPSC-2). We treated cells with 1 µM gemcitabine for 2 h and subjected cell pellets to LC–MS/MS analysis. Interestingly, the intracellular concentration of dFdCTP was comparable in M1 and M2-macrophages, BxPC3 and L3.6pl cells, but was higher in the hPSC-1 cell lines (Figure 3D), consistent with previously reported results in murine CAFs [10].

### 2.4. Pharmacological Depletion of TAMs Using Liposomal Clodronate Sensitizes KPC Tumors to Gemcitabine Treatment

**Figure 4:**
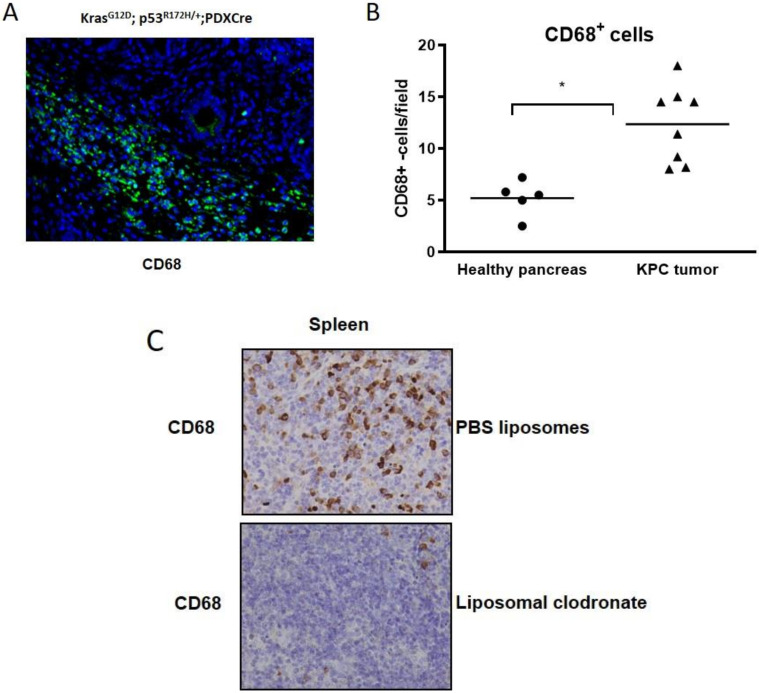

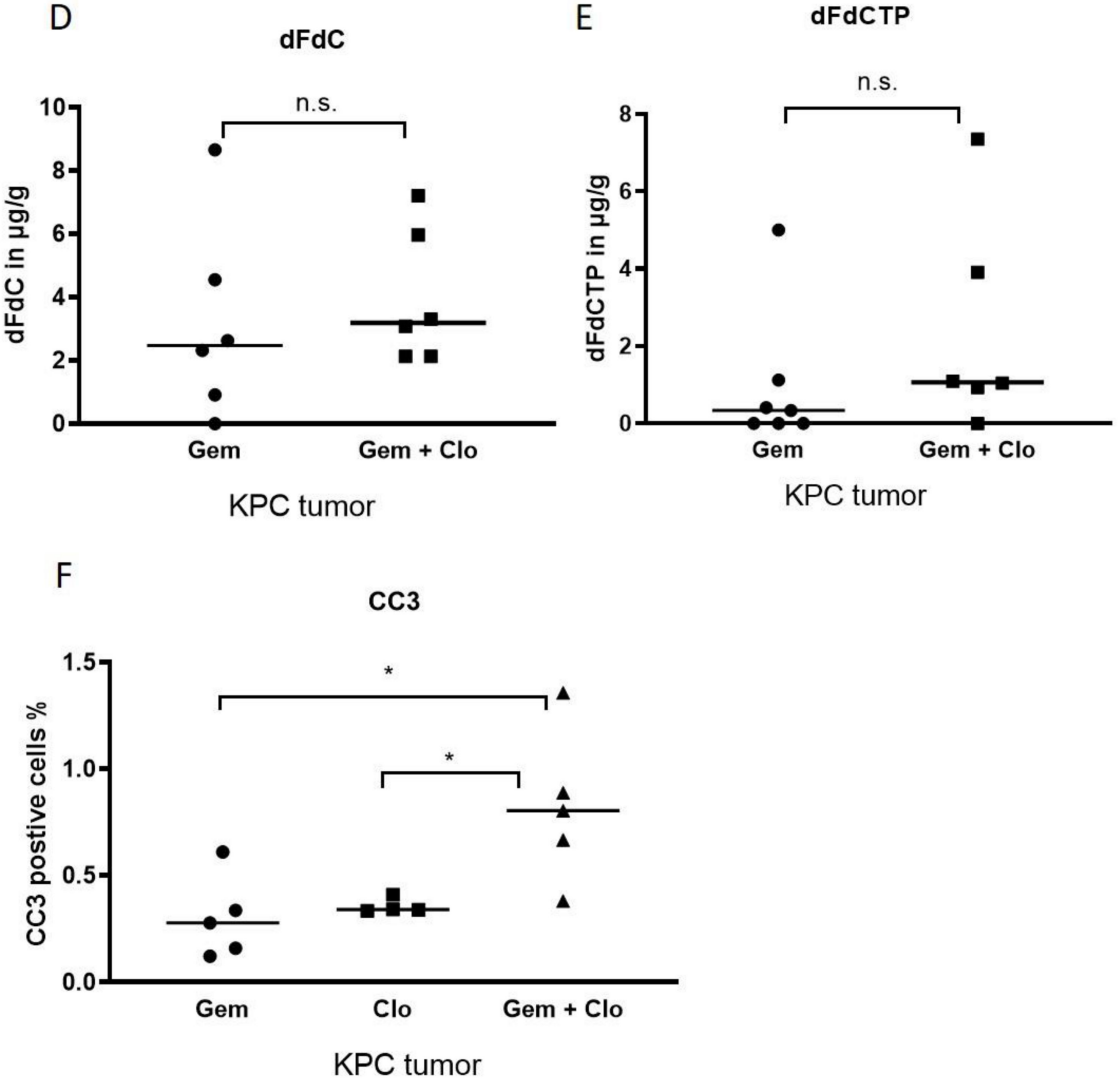
(**A**) Immunofluorescence of a KPC tumor showing dense infiltration of CD68+ macrophages (green) in the tumor microenvironment. (**B**) Manual quantification of CD68 positive cells in healthy pancreata (*n* = 5 mice) and KPC tumors (*n* = 8) reveals a significant increase in macrophages in tumor tissues (*p* < 0.002). (**C**) Representative immunohistochemistry for CD68 in splenic tissue showing robust depletion upon liposomal clodronate treatment. (**D**,**E**) Tumor biopsies from KPC mice treated with either gemcitabine (*n* = 6) or gemcitabine + liposomal clodronate (*n* = 6) were assessed for gemcitabine metabolites 2 h after the last injection of 100 mg/kg gemcitabine by LC–MS/MS. Native gemcitabine (dFdC) and the active form of gemcitabine 2’,2’-difluorodeoxyuridine-5’-triphosphate (dFdCTP) were not significantly altered between the two treatment cohorts. (**F**) CC3 immunohistochemistry reveals a significant increase in apoptotic cells in gemcitabine + liposomal clodronate treated KPC tumors compared to gemcitabine alone or liposomal clodronate (*p* < 0.01).

Since human and murine macrophages actively metabolized gemcitabine and significantly impeded the anti-neoplastic effects of gemcitabine on tumor cells in vitro, we hypothesized that pharmacological depletion of TAMs in vivo may synergize with gemcitabine treatment. To this end, we used the KPC mouse model that closely recapitulates the aggressive and desmoplastic nature of human PDAC. Immunohistochemical analysis revealed the robust infiltration of CD68+ macrophages within the TME of KPC mice that were significantly elevated compared to normal pancreas tissue (Figure 4A,B; *p* < 0.002).

To test the capacity of liposomal clodronate for CD68+ cell depletion, we administered the compound to healthy mice without a tumor burden (*n* = 4) and found the robust ablation of CD68+ splenic macrophages (Figure 4C).

Subsequently, we enrolled KPC mice that had developed pancreatic tumors, as evidenced by small animal ultrasound, and randomized them for treatment with either gemcitabine (100 mg/kg, three times per week), liposomal clodronate (70 mg/kg, bi-weekly) or a combination of liposomal clodronate and gemcitabine for 10 days (Supplementary Figure S4). At the endpoint, a final dose of gemcitabine was given 2 h prior to necropsy. As expected, the pharmacological depletion of TAMs by liposomal clodronate did not change the total drug levels of gemcitabine metabolites as evidenced by LC–MS/MS analysis of bulk tumor tissue (Figure 4D,E). However, immunohistochemistry revealed that the number of cleaved caspase-3 (CC3) positive tumor cells was significantly increased in endogenous tumor tissues that received the combination of gemcitabine and liposomal clodronate (Figure 4F), suggesting a shift of active gemcitabine metabolites towards proliferating tumor cells with subsequently increased apoptosis rates.

## 3. Discussion

PDAC is characterized by the accumulation of large amounts of ECM components such as collagen and hyaluronic acid, as well as abundant fibro-inflammatory cells that surround neoplastic cells at large numbers [8,27]. A highly debated question is whether this desmoplastic reaction in PDAC creates biophysical barriers for drug delivery or if cellular activities like enzymatic inactivation and scavenging play a more relevant role in the highly chemoresistant phenotype of human PDAC. Here, we revisit the role of the cellular TME, particularly macrophages as a barrier for gemcitabine, the front-line chemotherapy in PDAC using a previously established LC–MS/MS protocol. First, our in vivo pharmacokinetic studies reveal that bulk tumor biopsies accumulate significantly more gemcitabine metabolites than preneoplastic and normal pancreas tissue. This finding may be explained by the fact that tumor tissue is highly proliferative compared to normal pancreas tissue and PanINs. Furthermore, numerous cells within the TME actively metabolize and intracellularly store gemcitabine metabolites that are ultimately not available for tumor cells. Indeed, our in vitro data show that macrophages of human or murine origin actively metabolize gemcitabine resulting in a significant loss of cytotoxicity towards co-cultured tumor cells. This drug scavenging effect was recently described for CAFs and PSCs [10], but seems to be equally relevant for inflammatory cells. Using liposomal clodronate to deplete intratumoral TAMs, we could show improved efficacy of gemcitabine in the KPC model. Importantly, the amount of gemcitabine remained unchanged within in the bulk tumor suggesting an increased amount of cytotoxic gemcitabine that was not metabolized by macrophages and thus available to kill tumor cells. In line with our findings, an exciting study has recently shown that TAMs are programmed by PDAC cells to release deoxycytidine that competes with gemcitabine but not with other chemotherapies [18]. Therefore, our data support the hypothesis that the cellular TME creates biochemical barriers for gemcitabine reducing its efficacy in desmoplastic pancreatic tumors. Targeting of those cellular components, e.g., macrophages or CAFs, might be the most promising strategy to enhance efficacy of various chemotherapies in PDAC. However, TAMs possess tumor-promoting as well as tumor-suppressive functions, and broad depletion of TAMs for therapeutic purposes may also result in detrimental effects including suppression of resident or circulating immune cells [28]. Therefore, selective re-programming rather than depletion of TAMs might be the most appropriate therapeutic strategy in the future to avoid undesired immune suppressive or tumor-promoting effects.

Our study has several limitations. First, THP-1 cells are distinct from TAMs derived from PDAC. However, THP-1 cells can be easily grown and polarized in vitro, and thus represent an ideal tool for reproducible pharmacokinetic testing. Second, we use results from pharmacokinetic in vitro assays in order to explain the improved response to gemcitabine upon TAM depletion in vivo. However, as we cannot show direct gemcitabine scavenging of TAMs in KPC tumor tissue in vivo, it remains possible that the improved response to gemcitabine in combination with liposomal clodronate is achieved by biochemical sensitization of tumor cells upon TAM depletion rather than direct drug scavenging of TAMs. Moreover, we do not provide evidence that macrophage depletion results in more intracellular gemcitabine in tumor cells in vivo. Finally, survival studies using the combination of liposomal clodronate and gemcitabine in KPC mice would have been desirable but were not feasible due to the side effects of long-term liposomal clodronate administration in mice.

In summary, our current results underscore the fact that analysis of drug concentrations in desmoplastic bulk PDAC tissue is not appropriate to draw meaningful conclusions on the efficacy of the respective compound since the intratumoral uptake, distribution and metabolism of the various fibro-inflammatory cells is critical for therapeutic response. This may in fact explain the recent and past discrepancies between preclinical studies showing increased amounts of gemcitabine upon stromal depletion approaches [29,30], and subsequent clinical failure. In addition, our data may at also partially explain the difference between in vitro and in vivo efficacy of gemcitabine in PDAC and thus add to the growing body of evidence that therapeutic targeting of TAMs in PDAC might improve the therapeutic response by directly enhancing the available amount of intratumoral drug.

## 4. Materials and Methods

### 4.1. Genetically Engineered Mouse Models

The following genetically engineered mice were used for this study: *LSL-Kras^G12D^*;*p48-Cre* (KC) and *LSL-Kras^G12D^*; *LSL-Trp53^R172H^*;*Pdx-1-Cre* (KPC). KC mice develop acinar to ductal metaplasia (ADMs) and PanINs at an early age and slowly progress to advanced and metastatic PDAC after a long latency (usually >12 months), while KPC mice develop invasive and metastatic PDAC with 100% penetrance at an early age [23,31]. Both models recapitulate the full spectrum of histopathological and clinical features of human PDAC. All animal experiments were carried out using protocols approved by the Institutional Animal Care and Use Committee at the University Medical Center Göttingen. Mice were housed at a 12 h light, 12 h dark rhythm.

### 4.2. Therapeutic Intervention

KPC mice were subjected to treatment after detection of a pancreatic tumor of at least 0.5 cm in one dimension, using a Vevo2100 small animal ultrasound system as described before [32]. Treatment involved intraperitoneal injection of liposomal clodronate (twice per week, 70 mg/kg body weight), gemcitabine (twice per week, 100 mg/kg body weight) or a combination of both therapies. PBS-liposomes with an amount equivalent to liposomal clodronate was used as vehicle. For pharmacokinetic studies, KC mice were treated with gemcitabine (100 mg/kg body weight) once. Biopsies (*n* = 5) from tumor-bearing KC mice were partly overlapping with the KC-WT control group that was recently published from our group [4]. All tissues were harvested 2 h after the last gemcitabine dose for further analysis as previously described [21].

### 4.3. Liquid Chromatography–Mass Spectrometry/Mass Spectrometry (LC–MS/MS)

In cell culture experiments, 5 × 10^5^ cells were cultured and treated with 1 µM gemcitabine for 2 h or 24 h. After trypsinization, cell pellets were washed twice in cold PBS and stored at −80 °C. All experiments were performed in triplicate. Fresh frozen tumor samples and cell pellets were prepared and analyzed using LC–MS/MS for gemcitabine and metabolites as previously described [20]. LC–MS/MS was performed using a TSQ Vantage triple-stage quadrupole mass spectrometer (Thermo Fisher Scientific, Waltham, MA, USA) fitted with a heated electrospray ionization probe operated in positive and negative mode at a spray voltage of 2.5 kV, capillary temperature of 150 °C and vaporizer temperature of 250 °C. Quantitative data acquisition was done using LC Quan2.5.6 (Thermo Fisher Scientific, Waltham, MA, USA).

### 4.4. Drug Preparation

Gemcitabine hydrochloride (Sigma, St. Louis, MO, USA) was resuspended in sterile normal saline at 10 mg/mL. Clodronate liposomes and PBS liposomes were purchased from clodronateliposomes.org (Haarlem, The Netherlands). The preparation was described earlier [33]. For in vitro experiments, gemcitabine hydrochloride (Sigma, St. Louis, MO, USA), was used.

### 4.5. Cell Lines

The human pancreatic cancer cell lines BxPC3, L3.6pl, and the human monocyte cell line THP-1 were derived from ATCC and have been described before [34]. In addition 3 murine cell lines were derived from KPC tumors as previously reported, [31] and maintained in DMEM (Invitrogen, Carlsbad, CA, USA) +10% FBS (Thermo Fisher, Waltham, MA, USA). Human pancreatic stellate cells were used as previously described and labelled as hPSC-1 [35], and hPSC-2 [36].

### 4.6. Human Macrophage Polarization

Macrophage polarization was performed as described previously with little modifications [37]. In short, cells were seeded in a 6-well plate at a density of 1 × 10^6^/well or in a 6-cm dish at a density of 2.5 × 10^6^. To generate M1 polarized macrophages, cells were treated with 200 ng/mL phorbol 12-myristate 13-acetate (PMA, Sigma, St. Louis, MO, USA) for 6 h and then cultured with PMA plus 100 ng/mL lipopolysaccharide (LPS, Sigma, St. Louis, MO, USA) and 20 ng/mL interferon-γ (IF-γ, Peprotech, Cranbury, NJ, USA) for 66 h. To generate M2 polarized macrophages, cells were treated with 200 ng/mL PMA plus 20 ng/mL interleukin-4 (IL-4, Peprotech, Cranbury, NJ, USA) and 20 ng/mL interleukin-13 (IL-13, Peprotech, Cranbury, NJ, USA) for 72 h.

### 4.7. Murine Macrophage Isolation

Bone marrow derived macrophages (BMDMs) were isolated from mice with a C57BL/6 background as described earlier [38,39]. Briefly, we sacrificed the mice, isolated tibiae and femurs, cut the epiphyses and flushed the marrow using a syringe and a 25G needle. Then we centrifuged the bone marrow multiple times, using RPMI to wash it and an erythrocyte-lysis-buffer to eliminate the erythrocytes and their precursor cells. We seeded the cells yielding for 2 × 10^6^ cells/10 cm dish in RPMI and incubated them with 20 ng/mL recombinant murine M-CSF (Peprotech) for up to 7 days [40]. The resulting cells were predominantly macrophages and used for our experiments [40].

### 4.8. Culture with Conditioned Medium

M1-macrophages were generated through polarization of THP-1 cells. Murine macrophages were differentiated according to the protocol above. The macrophages were treated with culture medium containing gemcitabine in different concentrations or without a drug as control for 24 h. Cell viability was >80% at the time of medium collection compared to control. Subsequently, media were centrifuged at 1200 rpm for 3 min and conditioned medium supernatants collected.

### 4.9. Cell Viability Assays

Human and murine pancreatic cancer cells were seeded with 5000 cells/well on a 96 well plate and were allowed to attach for 24 h before adding conditioned medium and performing 72 h 3-[4,5-dimethylthiazol-2-yl]-2,5-diphenyltetrazolium bromide; thiazolyl blue (MTT) cell viability assay. Cells were treated with conditioned medium containing the GI_50_ of the respective tumor cell line. The control conditioned medium contained an equivalent dose of gemcitabine. Then, 72 h after treatment, MTT reagent (Thiazolyl Blue Tetrazolium Bromide, Sigma, St. Louis, MO, USA) was added to the media with a final concentration of 0.5 mg/mL and incubated for 2 h at 37 °C. Absorption was measured at 595 nm (PHOmo Microplate reader, Autobio Labtec Instruments, Zhengzhou, China). Cell viability was expressed relative to controls

### 4.10. Statistical Analysis

Data are presented as individual values or column bar graphs with the mean or median ± SEM. Statistical analysis was performed using GraphPad Prism 7.0 a using unpaired t-tests if not stated otherwise. *p* < 0.05 was considered statistically significant.

## Supplementary Materials

The following are available online at https://www.mdpi.com/2072-6694/12/7/1978/s1, Figure S1: MTT assay with conditioned media (CM) of human THP-1 cells or murine BMDM. Figure S2: MTT assay with conditioned media 24 h preincubated with gemcitabine or fresh gemcitabine. Figure S3: qRT-PCR for M1 and M2 markers in THP-1 macrophages. Figure S4: 10 days treatment schedule for tumor bearing KPC mice.
