# Supplementary material for: Depletion of Macrophages Improves Therapeutic Response to Gemcitabine in Murine Pancreas Cancer
Source: Cancers (Basel). 2020 Jul 20;12(7):1978. doi: 10.3390/cancers12071978 (PMC7409345; doi:10.3390/cancers12071978)
Supplement: Supplementary file 1 [file cancers-12-01978-s001.pdf]

# Suppl Figure 1

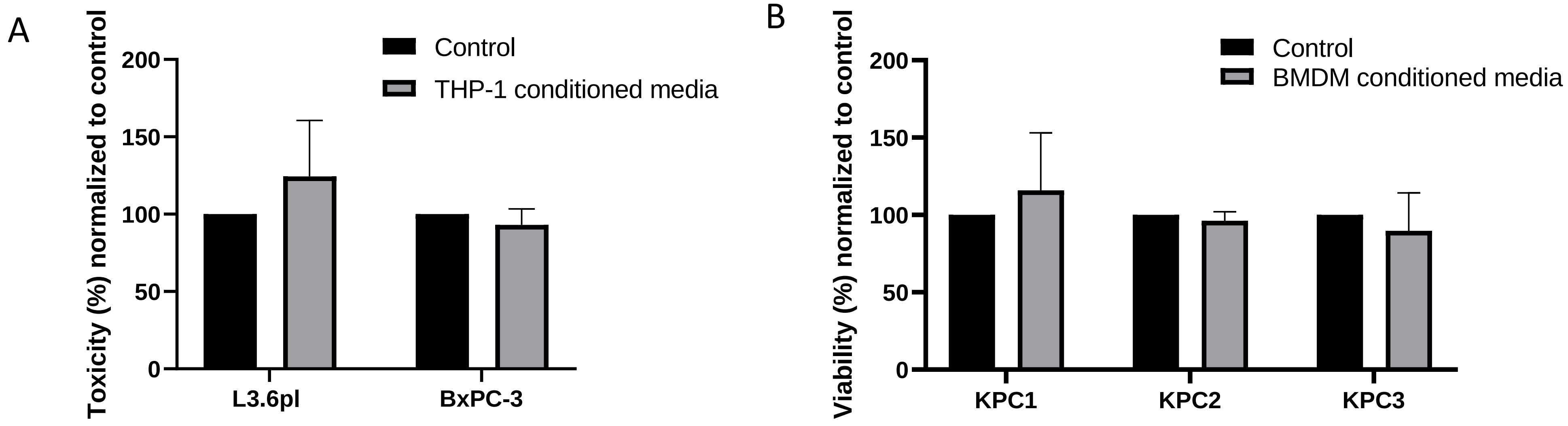


**A:** 72 hours MTT assay with conditioned media (CM) of human THP-1 cells and control media in 2 human pancreatic cancer cell lines L3.6pl and Bxpc3 shows no significant increase in cell viability. **B:** Equivalent assay with 3 murine KPC cell lines shows no change in cell viability in BMDM conditioned media compared to control media.

# Suppl Figure 2

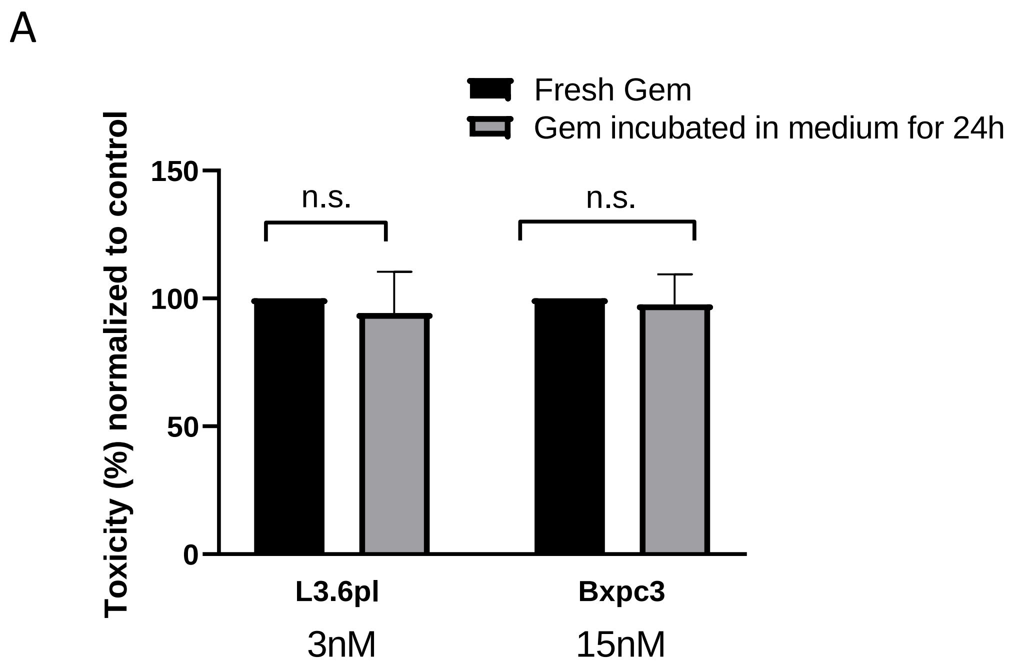


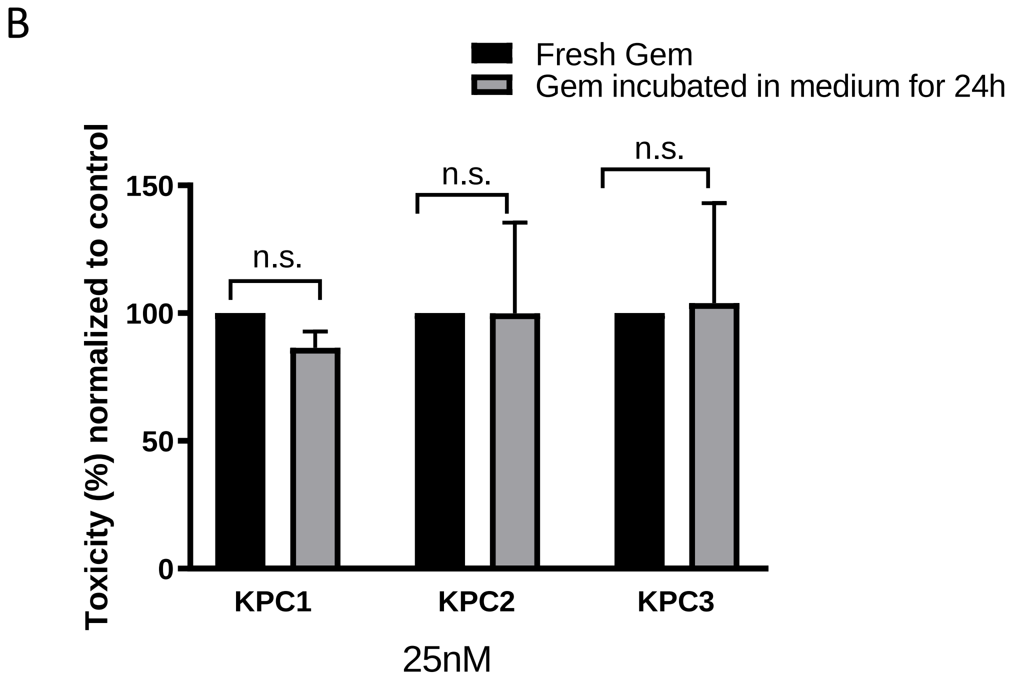


**A**: 72 hours MTT assay with conditioned media 24h preincubated with gemcitabine or fresh gemcitabine for L3.6pl and Bxpc3 shows no significant change in cytotoxicity. **B**: 72 hours MTT assay with conditioned media 24h preincubated with gemcitabine or fresh gemcitabine for murine KPC1-3 cell lines shows no significant change in cytotoxicity.

Suppl Figure 3

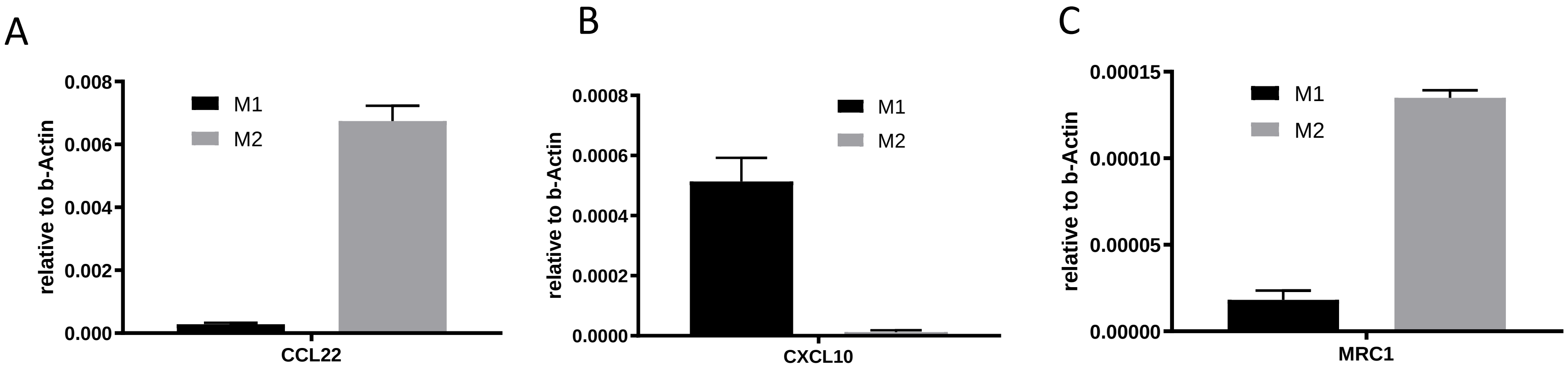


**A-C:** RNA isolated from THP-1 macrophages (M1, M2 polarization) were subjected to quantitative, reverse transcription-PCR and show distinct RNA expression of CCL22, CXCL10 and MRC1.

# Suppl Figure 4

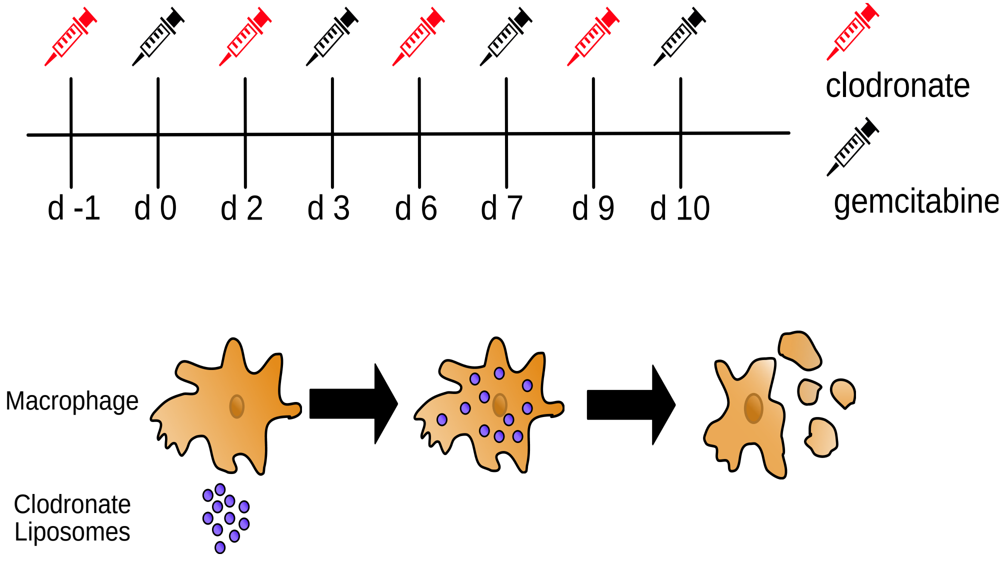


10 days treatment schedule for tumor bearing KPC mice.
